# Green tea polyphenol epigallocatechin-3-gallate inhibits advanced glycation end product-induced expression of tumor necrosis factor-α and matrix metalloproteinase-13 in human chondrocytes

**DOI:** 10.1186/ar2700

**Published:** 2009-05-15

**Authors:** Zafar Rasheed, Arivarasu N Anbazhagan, Nahid Akhtar, Sangeetha Ramamurthy, Frank R Voss, Tariq M Haqqi

**Affiliations:** 1 Department of Pathology, Microbiology, & Immunology, School of Medicine, University of South Carolina, 6439 Garners Ferry Rd, Columbia, SC 29209, USA; 2 Department of Orthopaedics, University of South Carolina, School of Medicine/Palmetto Richland Hospital, Two Medical Park, Columbia, SC 29203, USA

## Abstract

**Introduction:**

The major risk factor for osteoarthritis (OA) is aging, but the mechanisms underlying this risk are only partly understood. Age-related accumulation of advanced glycation end products (AGEs) can activate chondrocytes and induce the production of proinflammatory cytokines and matrix metalloproteinases (MMPs). In the present study, we examined the effect of epigallocatechin-3-gallate (EGCG) on AGE-modified-BSA (AGE-BSA)-induced activation and production of TNFα and MMP-13 in human OA chondrocytes.

**Methods:**

Human chondrocytes were derived from OA cartilage by enzymatic digestion and stimulated with *in vitro-*generated AGE-BSA. Gene expression of TNFα and MMP-13 was measured by quantitative RT-PCR. TNFα protein in culture medium was determined using cytokine-specific ELISA. Western immunoblotting was used to analyze the MMP-13 production in the culture medium, phosphorylation of mitogen-activated protein kinases (MAPKs), and the activation of NF-κB. DNA binding activity of NF-κB p65 was determined using a highly sensitive and specific ELISA. IκB kinase (IKK) activity was determined using an *in vitro *kinase activity assay. MMP-13 activity in the culture medium was assayed by gelatin zymography.

**Results:**

EGCG significantly decreased AGE-stimulated gene expression and production of TNFα and MMP-13 in human chondrocytes. The inhibitory effect of EGCG on the AGE-BSA-induced expression of TNFα and MMP-13 was mediated at least in part via suppression of p38-MAPK and JNK activation. In addition, EGCG inhibited the phosphorylating activity of IKKβ kinase in an *in vitro *activity assay and EGCG inhibited the AGE-mediated activation and DNA binding activity of NF-κB by suppressing the degradation of its inhibitory protein IκBα in the cytoplasm.

**Conclusions:**

These novel pharmacological actions of EGCG on AGE-BSA-stimulated human OA chondrocytes provide new suggestions that EGCG or EGCG-derived compounds may inhibit cartilage degradation by suppressing AGE-mediated activation and the catabolic response in human chondrocytes.

## Introduction

Osteoarthritis (OA), the most common form of arthritis, is a progressive degenerative joint disease that has a major impact on joint function and the patient's quality of life [1,2]. Many risk factors that contribute to disease onset have been identified, including systemic factors such as genetics, estrogen use, and bone density, and local biomechanical factors such as muscle weakness, obesity, and joint laxity [1]. The most important risk factor for OA besides female sex, obesity, and joint trauma is aging [1,2]. How aging contributes to the onset and progression of OA, however, is relatively unknown.

A prominent feature of aging is the modification of proteins by nonenzymatic glycation. Nonenzymatic glycation is a common post-translational modification of proteins caused by reducing sugars. The spontaneous condensation of reducing sugars with free amino groups in lysine or arginine residues on proteins leads to the formation of a reversible Schiff base, which is subsequently stabilized by Amadori rearrangement. The Maillard or browning reaction then converts the initially formed intermediate products into advanced glycation end products (AGEs) [3]. In addition to this classical pathway of AGE formation, it has recently been found that AGE formation can be initiated by metal-catalyzed glucose autooxidation as well as by lipid peroxidation (thereby providing an interesting link between lipid metabolism and the development of OA).

This diversity in reaction pathways results in a variety of chemical structures of AGEs. Some AGEs are adducts to proteins, while many others present protein–protein crosslinks. Once AGEs are formed, they cannot be removed from the protein; they only leave a tissue when the protein involved is degraded. Articular cartilage collagen has an exceptionally long half-life, and, since the rate of AGE accumulation is largely determined by the rate of protein turnover [4], this low turnover of cartilage constituents results in an abundant accumulation of AGEs in articular cartilage [5,6]. The accumulation of AGEs in cartilage leads to inferior mechanical properties [5,7] and to an alteration in cartilage metabolism [4,8]. More specifically, cartilage stiffness increases substantially with increasing AGE levels, and matrix synthesis by articular chondrocytes becomes impaired [5,7,9]. Accumulation of AGEs, however, is a proposed mechanism for the age-related development of OA [3,10]. Some studies also showed that still-healthy cartilage of patients with a focal degenerative cartilage lesion elsewhere in the joint has higher AGE levels than healthy cartilage from control individuals in which there are no signs of OA [11]. The age-related accumulation of AGE crosslinks presents a putative molecular mechanism whereby age contributes to the risk of developing OA. The accumulation of AGEs, however, is not only age related. AGE levels tend to be increased in diabetic patients, since the hyperglycemia accelerates AGE formation [12]. The correlation between diabetes mellitus and OA is supported by some older findings showing that radiographic OA is more common, more severe, and present earlier in patients with diabetes [13,14]. In addition, reports from more recent times still show a trend toward correlation of OA with diabetes [15]. OA therefore correlates with both aging and diabetes. In both aging and diabetes, AGE levels are increased. The levels of AGEs might therefore predict susceptibility to OA.

*In vivo *effects of AGEs on cartilage integrity have been reported in recent studies in beagle dogs and a canine model of OA induced experimentally by anterior cruciate ligament transection. Animals with elevated AGE levels had more severe OA than did those with normal AGE levels [10]. The mechanism by which AGEs influence cellular function in articular cartilage is poorly understood. The receptor for AGE (RAGE) is expressed in articular chondrocytes and synovial tissue macrophages of individuals with arthritis [16,17]. Activation of RAGE by multiple ligands including S100 protein, high-mobility group box chromosomal protein 1 and AGEs (complex and specific AGEs) in OA chondrocytes and synoviocytes results in increased production of various inflammatory mediators including TNFα and matrix metalloproteinase (MMP)-13 [18-20]. Previous studies have used complexes generated from BSA or a specific AGE, usually pentosidine or N3-carboxymethyllysine, to stimulate OA chondrocytes [21-23]. The AGEs used in the current study were produced by reacting endotoxin-free BSA with glycolaldehyde. The resulting AGE-BSA is a complex that includes N3-carboxymethyllysine, pentosidine, and other AGEs [24]. The results of the present study were therefore obtained with a complex of AGEs rather than with a particular AGE. MMPs, a large family of structurally related calcium-dependent and zinc-dependent proteolytic enzymes, are involved in the degradation of many different components of the extracellular matrix [17,25]. Both TNFα and MMPs are expressed in a number of different cell types and play a key role in diverse cellular processes, ranging from morphogenesis to tumor invasion to tissue remodeling [25,26]. Among the MMPs, MMP-13 (collagenase 3) is considered of particular interest due to its ability to digest type-2 collagen.

Green tea (*Camellia sinensis*), a popular beverage worldwide, has been shown to exert antimutagenic, antiproliferative, and anticarcinogenic effects, as well as anti-inflammatory activity in models of degenerative disorders [27-29]. We have earlier shown that green tea polyphenols inhibit the development of inflammatory arthritis in a mouse model [30] and that epigallocatechin-3-gallate (EGCG), the bioactive constituent of green tea, was nontoxic to human chondrocytes and inhibited the expression of inflammatory mediators *in vitro *[31-34]. The beneficial effects ascribed to drinking tea are believed to rely on the pharmacological actions of catechins, especially EGCG, and the derivatives of catechin components [35]. The effects of EGCG on AGE-induced damage in arthritis, however, remain to be elucidated. Since high levels of TNFα and MMPs are expressed and produced by AGE-activated human OA chondrocytes [18], in the present study we addressed for the first time the question of a possible inhibitory effect of EGCG on AGE-induced expression and production of TNFα and MMP-13 in OA chondrocytes. Our results showed that EGCG suppressed the AGE-induced TNFα and MMP-13 expression and production in OA chondrocytes, and that these effects were concomitant with inhibited activation of the mitogen-activated protein kinase (MAPK) subgroups p38-MAPK and JNK and the activation of the transcription factor NF-κB. Our results thus identify a unique mechanism of action of a dietary constituent and suggest that use of EGCG or compounds derived from it may have cartilage sparing effect in arthritis.

## Materials and methods

### Specimen selection and articular chondrocyte preparation

With Institutional Review Board approval, discarded cartilage samples were obtained from the knee joints of 13 OA patients aged 58 to 77 years (mean age, 66 ± 5.2 years; 11 female and two male Caucasians) who underwent joint replacement surgery at Palmetto-Richland Hospital, Columbia, SC, USA. The macroscopic cartilage degeneration was determined by staining of femoral head samples with India ink [36], and the cartilage with smooth articular surface (unaffected cartilage) was resected and used to prepare chondrocytes by enzymatic digestion as previously described [31-34,37]. Histological analysis of some of the unaffected cartilage samples was performed on 5-μm-thick sections stained with H & E and Safronin O and graded using the Mankin score [38]. Grading of the histology slides (data not shown) revealed that all of the cartilage pieces taken from the unaffected area had a Mankin score <2 for structure and a Mankin score of 1 for cellularity. Isolated chondrocytes were plated at a density of 1 × 10^6^/ml in 35-mm tissue culture dishes (Corning, NY, USA) in complete Ham's F-12 medium as previously described [31]. In some cases, OA chondrocytes passaged once were used.

### Preparation and characterization of AGE-BSA

AGE-BSA was prepared by reacting BSA (Sigma Chemical Co., St Louis, MO, USA) with glycoaldehyde (Sigma), according to the method described by Valencia and colleagues [21] with slight modifications. Briefly, endotoxin-free BSA (2 mg/ml) was incubated under nonreducing conditions with 70 mMfresh glycoaldehyde in PBS (pH 7.4) without calcium chloride and magnesium chloride for 3 days at 37°C. The reaction was terminated by removing nonreacted glycoaldehyde by dialyzing extensively against PBS.

Characterization of AGE-BSA was performed as previously described in detail [21-23]. Briefly, the AGE-specific absorbance was read at 340 nm [21] and AGE-specific fluorescence was detected at excitation/emission wavelengths of 360/430 nm [22], 330/395 nm, 365/440 nm, 485/530 nm, 280/350 nm and band widths set at ex40/em40 [23]. All fluorescence data are given normalized to the corresponding control. Absorbance and fluorescence were read using the Synergy HT microplate reader (Biotek Instruction, Winooski, VT, USA). The UV-visible absorption spectra of native BSA and of AGE-BSA were recorded on a Shimadzu UV-1800 Spectrophotometer. The electrophoretic migration of native and modified BSA samples was analyzed by reducing SDS-PAGE on 10% polyacrylamide gel with 4% stacking gel [39].

### Treatment of chondrocytes with AGE-BSA and EGCG

We first determined whether AGE-BSA and EGCG (purity ≥ 95%, catalogue number E4143; Sigma) affect the viability of OA chondrocytes *in vitro*. Human OA chondrocytes (1 × 10^6^/ml) were plated in 35-mm culture dishes (Becton-Dickinson, Franklin Lakes, NJ, USA) in complete Ham's F-12 medium and serum-starved for 12 hours/overnight. Chondrocytes were treated with various doses of AGE-BSA (20 to 600 μg/ml) and EGCG (25 to 200 μM), and after 24 hours the incubation cytotoxicity of AGE-BSA and EGCG was examined using the CytoTox-Glo™ Cytotoxicity Assay Kit (Promega, Madison, WI, USA). Primary chondrocytes were pretreated with different doses of EGCG for 1 or 2 hours prior to stimulation with AGE-BSA. Chondrocytes cultured without AGE-BSA or EGCG served as controls. All experiments were performed within 4 days of the primary culture to avoid dedifferentiation of OA chondrocytes.

### Quantitative real-time PCR

Real-time quantitative PCR was used to quantify the expression of mRNA for TNFα and MMP-13 with expression of GAPDH as endogenous control. Total RNA was separated from OA chondrocytes by the Quick Gene automated system according to the manufacturer's instruction (Quick Gene, Holliston, MA, USA). First-strand cDNA was synthesized using 1 μg total RNA and the SuperScript First Strand cDNA synthesis kit (Invitrogen, Carlsbad, CA, USA). Primers used for PCR assisted amplification were: TNFα (NM_000595: forward, 5'-AGG ACG AAC ATC CAA CCT TCC CAA-3'; reverse, 5'-TTT GAG CCA GAA GAG GTT GAG GGT-3'), MMP-13 (NM_002427: forward, 5'-CGC CAG AAG AAT CTG TCT TTA AA-3'; reverse, 5'-CCA AAT TAT GGA GGA GAT GC-3'), and GAPDH (NM_002046.3: forward, 5'-TCG ACA GTC AGC CGC ATC TTC TTT-3'; reverse, 5'-ACC AAA TCC GTT GAC TCC GAC CTT-3'). PCR amplification was carried out using the core kit for SYBR Green (Applied Biosystems, Foster City, CA, USA) and the Step One Real Time PCR System (Applied Biosystems). Typical profile times used were an initial step of 95°C for 10 minutes, followed by a second step at 95°C for 15 seconds and 60°C for 60 seconds for 40 cycles with melting curve analysis. The level of target mRNA was normalized to the level of GAPDH and compared with control (untreated sample). Data were analyzed using the ΔΔCT method [40].

### RT-PCR

To analyze the transcription of type-2 collagen (COL2A1), type-10 collagen (COL10A1), aggrecan (ACAN), and SRY-box containing gene 9 (SOX-9), total cellular RNA was prepared using a commercially available kit (Qiagen, Valencia, CA, USA). First-strand cDNA was synthesized using 1 μg total RNA and the SuperScript First Strand cDNA synthesis kit (Invitrogen). cDNAs of COL2A1, COL10A1, ACAN, and SOX-9 were used as positive controls in the PCR reaction and were generously provided by Dr Thomas Herring (Department of Orthopaedics, Case Western Reverse University School of Medicine, Cleveland, OH, USA). RT-PCR was carried out utilizing the PTC-100™ Peltier Thermal Cycler (MJ Research, Ramsey, MN, USA). Primers used for PCR-assisted amplification were: COL2A1 (NM_001844: forward, 5'-ACG TGA AAG ACT GCC TCA GC-3'; reverse, 5'-TTT CAT CAA ATC CTC CAG CC-3'; expected size of the DNA fragment, 352 bp), COL10A1 (NM_000493: forward, 5'-TGA TCC TGG AGT TGG AGG AC-3'; reverse, 5'-GAG ATC GAT GAT GGC ACT CC-3'; expected size of the DNA fragment, 703 bp); ACAN (NM_013227: forward, 5'-GAC CTG CAA GGA GAC AGA GG-3'; reverse, 5'-CCA CTG GTA GTC CTT GGG CAT-3'; expected size of the DNA fragment, 256 bp), and SOX-9 (NM_011448: forward, 5'-GAT TTT TCA CGC AGC CCT AA-3'; reverse, 5'-ATA CAG TCC AGG CAG ACC CA-3'; expected size of the DNA fragment, 637 bp). The reaction was cycled according to the following scheme: 40 minutes at 72°C, followed by 40 cycles of 1 minute at 95°C, 1 minute at 60°C, and 2 minutes at 72°C, followed by a final 15-minute extension. The amplified products were electrophoresed on 1.2% agarose gels in TAE buffer and were visualized by ethidium bromide staining.

### Cytokine ELISA

Briefly, OA chondrocytes were stimulated with AGE-BSA (600 μg/ml) for 24 hours with or without pretreatment with EGCG. TNFα present in the culture medium was quantified using the TNFα-specific ELISA according to the manufacturer's instructions (R&D Systems, Minneapolis, MN, USA). Plates were read at 450 nm using a Synergy HT microplate reader (Biotek Instrument, Winooski, VT, USA).

### Western immunoblotting

Stimulated and control chondrocytes were washed with cold PBS and lysed using the previously described cell lysis buffer (50 mM Tris-HCl, pH 7.4; 150 mM NaCl; 1% Triton X-100; 0.1% SDS; 0.5% sodium deoxycholate; 1 mM EDTA; 1 mM EGTA; Complete^® ^protease and phosphatase inhibitors) [41]. Cytoplasmic and nuclear fractions were prepared as previously described [42]. Total lysate or nuclear/cytoplasmic fraction protein (45 μg/lane) or concentrated cell culture supernatant was resolved by SDS-PAGE (10% resolving gel with 4% stacking) and was transferred to nitrocellulose membranes (Bio-Rad, Hercules, CA, USA). Membranes were blocked with blocking buffer containing nonfat dry milk powder in Tris-buffered saline and 0.1% Tween-20. Blots were probed with 1:200 to 1:1,000 diluted primary antibodies specific for the target protein. Immunoreactive proteins were visualized using 1:1,000 diluted horseradish peroxidase-linked secondary antibodies and enhanced chemiluminescence (GE Healthcare, Milwaukee, WI, USA) [43]. Images were captured using AFP-Imaging System (Minimedical Series, Elms Ford, NY, USA). Anti-MMP-13 antibody (sc-30073) was purchased from Santa Cruz Biotechnology (Santa Cruz, CA, USA), anti-NF-κB p65 antibody (IMG-150) was from Imgenex (San Diego, CA, USA), and anti-IκBα antibody (#9242), anti-phospho-p38 MAPK antibody (#9215S), anti-p38 MAPK antibody (#9212), anti-phospho-SAPK/JNK antibody (#9251S), anti-SAPK/JNK antibody (#9252), anti-phospho ERK P44/42 MAPK antibody (#9101S) and anti-ERK P44/42 MAPK antibody (#9102) were from Cell Signaling Technology (Amherst, Beverly, MA, USA).

### Gelatin zymography

Gelatin zymography was performed essentially as previously described [41]. Briefly, an equal volume of cell culture supernatant was mixed with nonreducing sample buffer (4% SDS, 0.15 M Tris (pH 6.8), and 20% (volume/volume) glycerol containing 0.05% (weight/volume) bromophenol blue) and resolved on a 10% polyacrylamide gel containing copolymerized 0.2% gelatin (Bio-Rad). Commercially available MMP-13 (catalogue number PF094; EMD Chemicals, Gibbstown, NJ, USA) was used as a positive control. The product used was supplied as concentrated conditioned medium, and 2.5 μl was used. After electrophoresis, gels were washed twice, for 15 minutes each time, with 2.5% Triton X-100. Digestion was carried out by incubating the gel in the gelatinase buffer (50 mM Tris-HCl (pH 7.6), 10 mM CaCl_2_, 50 mM NaCl, and 0.05% Brij-35) at 37°C for 16 hours. The gel was stained with 0.1% Coomassie brilliant blue R350 (GE Healthcare, Piscataway, NJ, USA), and the locations of gelatinolytic activity were revealed as clear bands on a background of uniform light blue staining. Electrophoretic migration of MMP-13 in the supernatant was compared with known molecular weight standards and also with clear bands of MMP-13 activity produced by the positive control. After development, gel images were captured and the clear bands were analyzed using Un-Scan-It software (Silk Scientific Corporation, Orem, UT, USA).

### NF-κB DNA binding activity assay

Activation of NF-κB p65 in human OA chondrocytes pretreated with EGCG and then stimulated with AGEs was determined using a highly sensitive and specific Transcription Factor ELISA Kit according to the instructions of the manufacturer (catalogue number EK1121; Panomics, Fremont, CA, USA). The color intensity was read at 450 nm using the Synergy HT ELISA plate reader (Biotek).

### *In vitro *IKKβ kinase assay

The effect of EGCG on purified IκB kinase (IKK) activity was determined with a highly sensitive HTScan^® ^IKKβ Kinase Assay Kit according to the instructions of the manufacturer (catalogue number 7549; Cell Signaling Technology). The kit provides a means of performing kinase activity assays with recombinant human IKKβ kinase. It includes active IKKβ kinase (supplied as GST fusion protein), a biotinylated peptide substrate, and a phospho-serine antibody for detection of the phosphorylated form of the substrate peptide. Purified IKKβ kinase was pretreated with different doses of EGCG (5 to 200 μM) 5 minutes prior to the treatment of substrate peptide. The assay was performed on a 96-well high-binding streptavidin-coated plate, and the absorbance (*A*) of each well was read at 450 nm using the Synergy HT ELISA plate reader (Biotek). Each kinase assay was performed in triplicate. The percentage inhibition of IKKβ kinase activity was calculated using the formula:



### Densitometric analysis

Measurements of the scanned bands were performed using Un-Scan-It software. Each band was scanned five times, and the mean band intensity (pixels per band) was obtained. Data were normalized to suitable loading controls and expressed as the mean ± standard deviation followed by appropriate statistical analysis.

### Statistical analysis

All measurements were performed in duplicate and were repeated at least three times using different age-matched and sex-matched OA samples. All statistical analyses were performed using Origin 6.1 software package (one-paired two-tailed *t *test with one-way analysis of variance and Tukey's *post-hoc *analysis) and *P *< 0.05 was considered significant. Values shown are the mean ± standard error of the mean unless stated otherwise.

## Results

### Characterization of AGE-BSA

**Figure 1:**
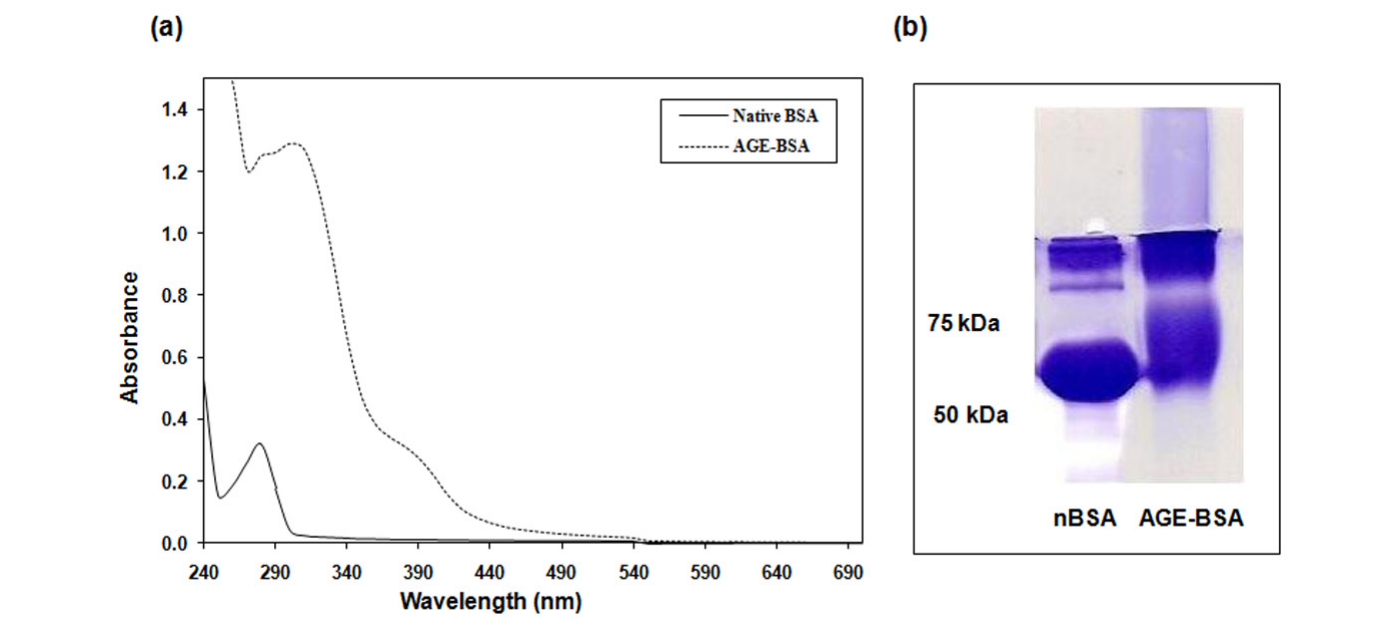
Absorbance spectra and electrophoresis of native BSA and advanced glycation end product-BSA. **(a) **Absorbance spectra of native BSA and advanced glycation end product (AGE)-BSA. Samples were incubated with or without glycoaldehyde in PBS, pH 7.2, with equal protein concentrations. **(b) **Electrophoresis of native BSA and AGE-BSA. Samples were electrophoresed using 10% SDS-PAGE with 4% stacking gel. The gel was run for 1.5 hours at 125 V. The precision plus protein standard (Bio-Rad) served as the molecular size marker. AGE-BSA was derived from the reaction between endotoxin-free BSA (2 mg/ml) and glycoaldehyde (70 mM).

Glycoaldehyde-induced modifications of BSA were studied by UV-visible absorption spectroscopy, gel electrophoresis, and AGE-specific fluorescence. The absorption spectra of AGE-BSA (AGEs) revealed a marked UV hyperchromicity (74.4%) at 280 nm (Figure 1a). For further characterization of *in vitro*-produced AGEs, samples were analyzed by SDS-PAGE under reducing conditions. An accompanying decrease in the electrophoretic mobility and smear towards higher molecular size ranges from 65 to 98 kDa was noted, indicating a higher level of glycoaldehyde-mediated modifications (Figure 1b). AGE-specific absorbance was detected at 340 nm [21] and was found to be significantly high in glycoaldehyde-treated BSA as compared with native BSA (*P *< 0.001). AGE-specific fluorescence was detected at the excitation/emission wavelengths described elsewhere [22,23] and were found to be very high for glycoaldehyde-treated BSA compared with native BSA, signifying the presence of a higher level of modifications. Characterization of AGE-specific modifications on BSA resulting from incubation with glycoaldehyde is summarized in Table 1.

**Table 1:** Characterization of native BSA and AGE-BSA under identical experimental conditions

Parameter	AGE-BSA	Native BSA (control)
*A *_340_	0.625 ± 0.13*	0.228 ± 0.05
Fluo [ex 360/em 430 nm]	749.4 ± 19.5**	80.4 ± 4.8
Fluo [ex 330/em 395 nm]	754.6 ± 17.2**	83.2 ± 5.7
Fluo [ex 365/em 440 nm]	741.4 ± 28.1**	84.8 ± 5.2
Fluo [ex 485/em 530 nm]	734.1 ± 20.4**	85.1 ± 5.3
Fluo [ex 280/em 350 nm]	733.2 ± 19.1**	84.0 ± 4.4

Data expressed as the mean ± standard deviation of 12 independent assays. *A*_340_, absorbance at 340 nm; AGE, advanced glycation end product; Fluo [ex/em], fluorescence [excitation/emission], Band width was set at 40/40 for fluorescence measurement. *P < 0.01 versus control, **P < 0.0001 versus control.

### Human chondrocytes in monolayers maintain their chondrogenic phenotype

**Figure 2:**
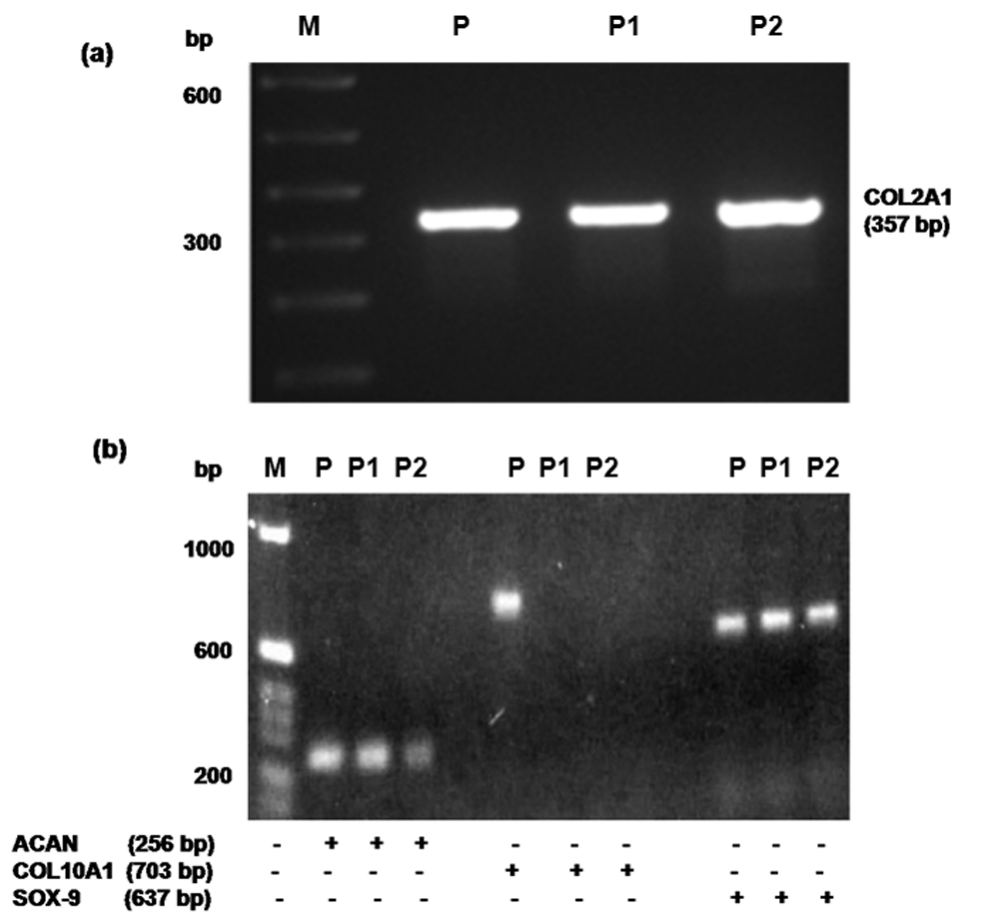
Human osteoarthritis chondrocytes in monolayer culture maintain their phenotype. Primary chondrocytes from osteoarthritis patients were cultured for 72 hours and were then split and cultured for an additional 3 days (passage 1). Expression of **(a) **type-2 collagen (COL2A1) and **(b) **aggrecan (ACAN), type-10 collagen (COL10A1) and SRY-box containing gene 9 (SOX-9) was determined by RT-PCR. M, 100 bp DNA ladder; P, positive control cDNA; P1, primary chondrocytes; P2, passage 1 chondrocytes.

We determined whether primary human OA chondrocytes and chondrocytes passaged once used in these studies maintained their phenotype – by analyzing the expression of type-2 collagen, aggrecan, and SOX-9 mRNA, which are considered to be signatures of the chondrogenic phenotype [44]. Our results show that primary or passage-1 human OA chondrocytes in monolayer culture maintained their phenotype, when they were plated (1 × 10^6^/ml) in 35-mm culture dishes in complete Ham's F-12 medium with 10% FCS and were allowed to grow at 37°C and 5% CO_2 _in a tissue culture incubator, as judged by the continued expression of COL2A1 (Figure 2a), aggrecan, and SOX-9 mRNAs, whereas COL10A1 mRNA was not expressed (Figure 2b). Based on these data, OA chondrocytes were used within 72 hours after plating to avoid possible dedifferentiation.

### Induction of TNFα and MMP-13 expression by AGE-BSA in human osteoarthritis chondrocytes

OA chondrocytes were treated with increasing doses of AGE-BSA (20 to 600 μg/ml) and the mRNA expression of TNFα and MMP-13 was determined using a highly sensitive and specific quantitative RT-PCR method. We found AGE-BSA dose-dependently induced TNFα and MMP-13 mRNA expressions, and the maximum stimulation of chondrocytes was found to be at 600 μg/ml AGE-BSA (data not shown). Based on these data, we used a concentration of 600 μg/ml AGE-BSA for stimulation of chondrocytes in all of our experiments.

### Effect of EGCG on AGE-BSA-induced expression and production of TNFα in osteoarthritis chondrocytes

**Figure 3:**
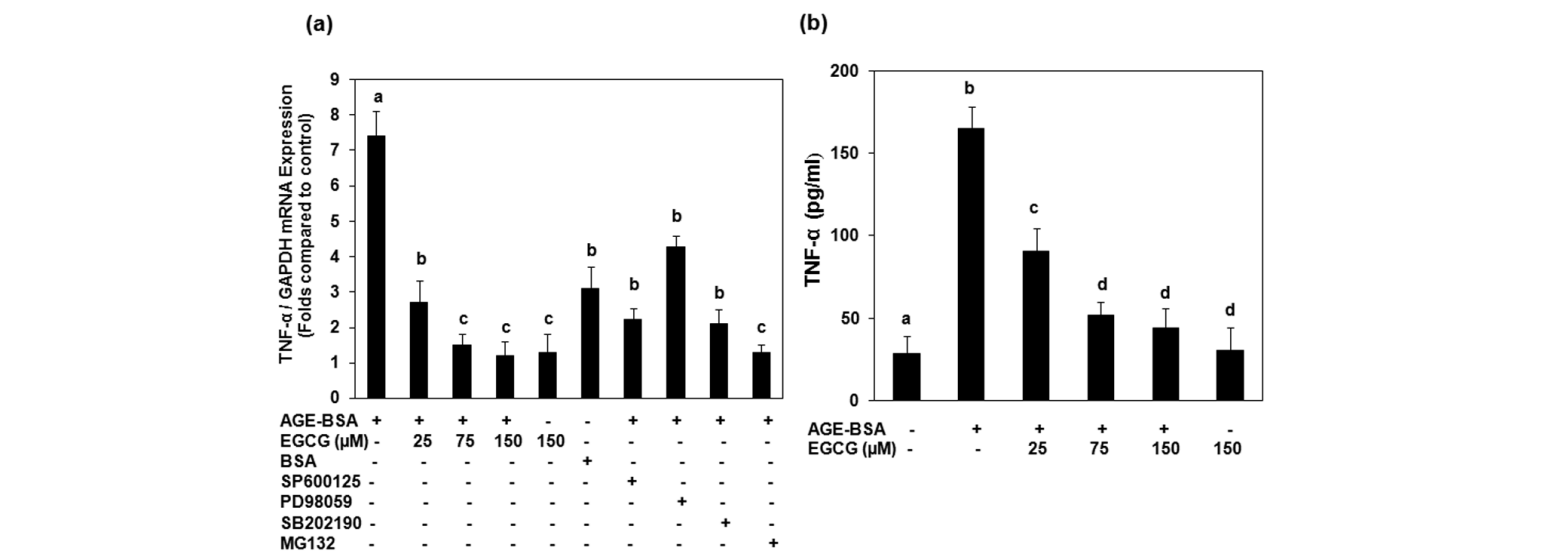
Gene expression and production of TNFα in advanced glycation end product-BSA-stimulated osteoarthritis chondrocytes. **(a) **Effect of epigallocatechin-3-gallate (EGCG), specific inhibitors for mitogen-activated protein kinases and NF-κB on the gene expression of TNFα in advanced glycation end product (AGE)-BSA-stimulated osteoarthritis (OA) chondrocytes. Primary chondrocytes were pretreated with EGCG (25 to 150 μM) for 2 hours and were stimulated by AGE-BSA (600 μg/ml) for 8 hours. Folds of TNFα mRNA expression, as compared with control and normalized to GAPDH, were determined by quantitative RT-PCR. Concentrations of specific inhibitors of JNK (SP600125), ERK (PD98059), p38 (SB202190) and NF-κB (MG-132) used in these studies were 10 μM, 50 μM, 100 μM and 100 μM, respectively. Native BSA (600 μg/ml) was used as a negative control. **(b) **Effect of EGCG on the production of TNFα in AGE-BSA-stimulated OA chondrocytes. Primary chondrocytes were pretreated with EGCG (25 to 150 μM) for 2 hours and were stimulated by AGE-BSA (600 μg/ml) for 24 hours. The production level of TNFα was determined by sandwich ELISA. Results are representative (mean ± standard error of the mean) of duplicate experiments with OA chondrocytes obtained from five age-matched and sex-matched OA donors; data without a common letter differ, *P *< 0.05.

OA chondrocytes (80% confluent) were pretreated with EGCG (25 to 150 μM) for 2 hours, and were then stimulated with *in vitro-*generated AGE-BSA (600 μg/ml) for 8 hours. No cytotoxic effect of EGCG was noted at the dose used (data not shown). The level of TNFα mRNA was quantified by a highly sensitive and specific quantitative RT-PCR method, and values were compared with control. Our results showed that OA chondrocytes treated with AGE-BSA had a higher level of TNFα mRNA compared with unstimulated OA chondrocytes. TNFα mRNA levels showed a marked decline, however, in the samples pretreated with EGCG and then stimulated with AGE-BSA (Figure 3a). To determine whether inhibition of gene expression also affected the protein level, culture supernatants were assayed for TNFα protein using TNFα-specific ELISA. As shown in Figure 3b, pretreatment with 25 to 150 μM EGCG significantly decreased the AGE-BSA-induced TNFα production in the culture supernatant of AGE-BSA-stimulated OA chondrocytes. It is also to be noted that maximum suppression was observed in cultures treated with 75 μM EGCG, after which no further decline was found (Figure 3b).

### EGCG downregulated the expression and production of MMP-13 in AGE-BSA-stimulated osteoarthritis chondrocytes

**Figure 4:**
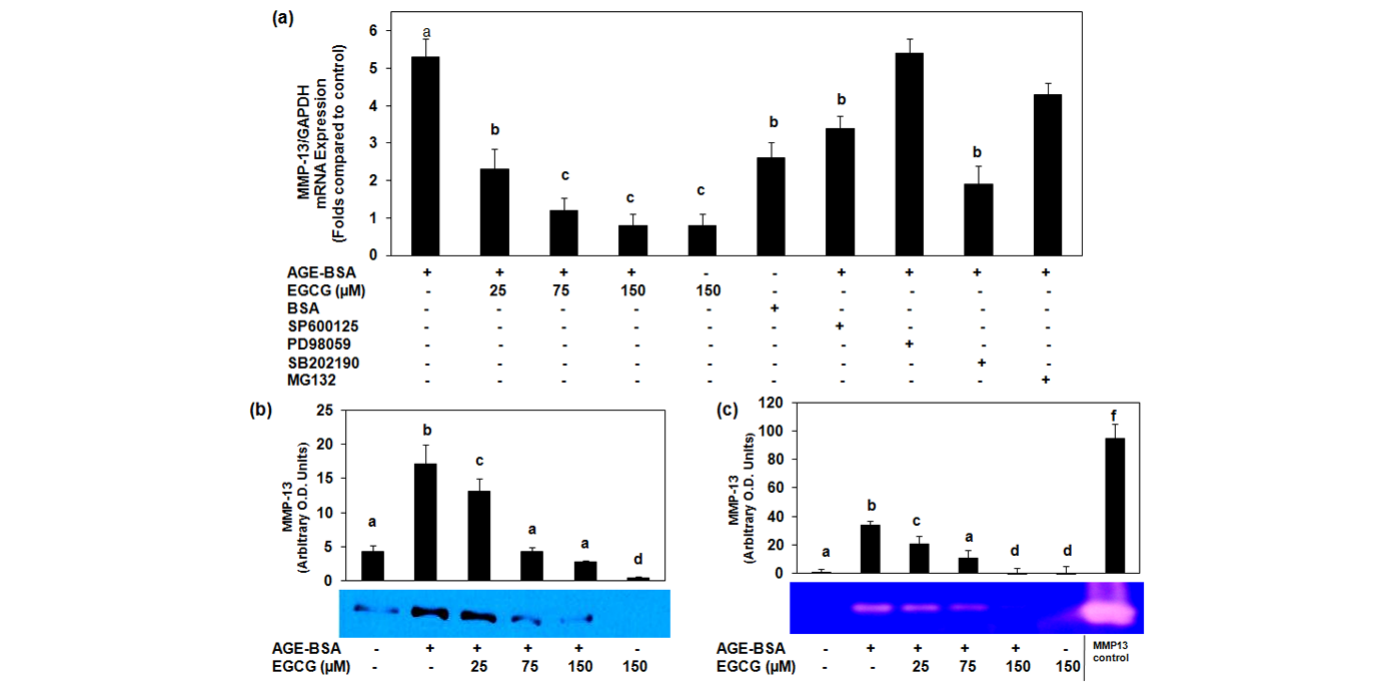
Gene expression and production of matrix metalloproteinase-13 in advanced glycation end product-BSA-stimulated osteoarthritis chondrocytes. **(a) **Effect of epigallocatechin-3-gallate (EGCG), specific inhibitors for mitogen-activated protein kinases and NF-κB on the gene expression of matrix metalloproteinase (MMP)-13 in advanced glycation end product (AGE)-BSA-stimulated osteoarthritis (OA) chondrocytes. Primary human chondrocytes were pretreated with EGCG (25 to 150 μM) for 2 hours and were stimulated with AGE-BSA (600 μg/ml) for 8 hours. Expression of MMP-13 mRNA was normalized to GAPDH and compared with the levels present in control. Concentrations of specific inhibitors of JNK (SP600125), ERK (PD98059), p38 (SB202190) and NF-κB (MG-132) used in these studies were 10 μM, 50 μM, 100 μM and 100 μM, respectively. Native BSA (600 μg/ml) was used as negative control. Results are representative (mean ± standard error of the mean) of duplicate experiments with chondrocytes obtained from five age-matched and sex-matched OA donors; data without a common letter differ, *P *< 0.01. **(b), (c) **Effect of EGCG on the production of MMP-13 in AGE-BSA-stimulated OA chondrocyte culture medium. Primary chondrocytes were pretreated with EGCG (25 to 150 μM) for 2 hours and were stimulated with AGE-BSA (600 μg/ml) for 24 hours. MMP-13 production was analyzed in cell culture supernatant by (b) western blotting and (c) gelatin zymography. Equal volumes of culture supernatant were loaded on polyacrylamide gel. The MMP-13 positive control (EMD Chemicals) was also used. Band images were digitally captured and the band intensities (pixels/band) were obtained using the Un-Scan-It software and are expressed in arbitrary optical density units. Data shown are cumulative of two experiments. OD values presented as mean ± standard deviation; data without a common letter differ, *P *< 0.05.

Pretreatment of OA chondrocytes with EGCG inhibited AGE-BSA-induced gene expression of MMP-13 in a dose-dependent manner as determined by quantitative RT-PCR (Figure 4a). Again the maximum inhibitory effect of EGCG was found at 75 μM concentration. We also determined the effect of EGCG on the production of MMP-13 in the culture supernatant by western immunoblotting using anti-MMP-13 antibody (Figure 4b). Analysis of the immunoblot revealed that the levels of MMP-13 were high in the supernatant of chondrocytes treated with AGE-BSA when compared with the levels detected in untreated chondrocytes culture, where MMP-13 was barely detectable. Importantly, the AGE-BSA-stimulated increase in MMP-13 expression was inhibited by EGCG to less than the basal level (Figure 4b). These results were further verified using gelatin zymography (Figure 4c). Zymographic analysis showed that OA chondrocytes stimulated with AGE-BSA had enhanced levels of active MMP-13 compared with the levels detected in control OA chondrocytes. Importantly, chondrocytes pretreated with different doses of EGCG and then stimulated with AGE-BSA showed reduced levels of active MMP-13 in the culture supernatant. Taken together these data indicate that the AGE-BSA-induced production of active MMP-13 was inhibited by EGCG in human OA chondrocytes (Figure 4c).

### Effect of EGCG on the activation of MAPKs in AGE-BSA-stimulated osteoarthritis chondrocytes

**Figure 5:**
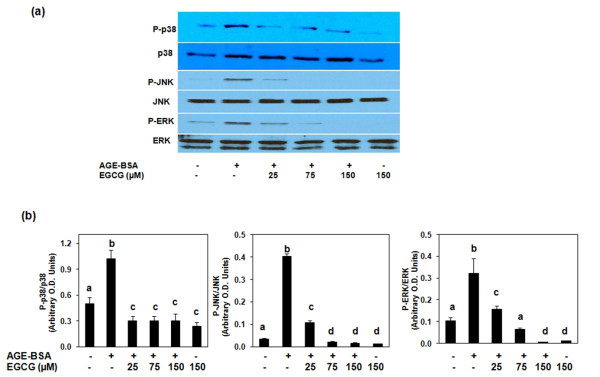
Mitogen-activated protein kinase phosphorylation in advanced glycation end product-BSA-stimulated osteoarthritis chondrocytes. **(a) **Effect of epigallocatechin-3-gallate (EGCG) on mitogen-activated protein kinase phosphorylation in advanced glycation end product (AGE)-BSA-stimulated osteoarthritis (OA) chondrocytes. After pretreatment with EGCG (25 to 150 μM) for 1 hour at 37°C, primary human chondrocytes (70 to 80% confluent) were incubated with AGE-BSA (400 μg/ml) for 45 minutes, and then the phosphorylation of p38, JNK, and ERK was determined by western blot analysis. **(b) **Band images were digitally captured and the band intensities (pixels/band) were obtained using the Un-Scan-It software and are expressed in arbitrary optical density units. Data shown are cumulative of two experiments. OD values presented as mean ± standard deviation; data without a common letter differ, *P *< 0.05.

Activation of MAPKs is intimately associated with the expression of proinflammatory mediators [26]. To determine whether the inhibition of TNFα and MMP-13 expressions was due to EGCG-mediated inhibition of the MAPK pathway, we examined the effect of EGCG on the activation of MAPKs in AGE-BSA-stimulated human OA chondrocytes. OA chondrocytes were pretreated with EGCG (25 to 150 μM) for 1 hour and then stimulated with AGE-BSA for 45 minutes, and the cell lysate was analyzed by western immunoblotting. Pretreatment of chondrocytes with EGCG attenuated the AGE-BSA-induced phosphorylation of p38-MAPK, JNK-MAPK and to a lesser extent of ERK-MAPK (Figure 5a,b). To further strengthen the relation of p38, JNK and ERK inhibition by EGCG and proinflammatory cytokine TNFα and MMP-13 expressions in AGE-BSA-stimulated human OA chondrocytes, we used pharmacological agents that inhibit p38-, JNK- and ERK-MAPKs.

Treatment of OA chondrocytes with the selective p38 inhibitor SB202190 (100 μM), the JNK inhibitor SP600125 (10 μM), and the ERK inhibitor PD98059 (50 μM) blocked the AGE-BSA-induced TNFα mRNA expression as determined by quantitative RT-PCR, but the effect was more pronounced in the case of p38-MAPK and JNK (Figure 3a). AGE-BSA-induced MMP-13 mRNA expression was significantly inhibited by SB202190 and SP600125 (*P *< 0.05), but the affect was more pronounced when SB202190 was used. Pretreatment of OA chondrocytes with PD98059 had no effect on MMP-13 mRNA expression in AGE-BSA-stimulated OA chondrocytes (Figure 4a). These data support the contention that inhibition of AGE-BSA-induced TNFα (Figure 3a) and MMP-13 (Figure 4a) expressions by EGCG in OA chondrocytes was mediated, at least in part, by the inhibition of AGE-induced activation of the p38-MAPK and JNK-MAPK pathways.

### Effect of EGCG on NF-κB activation in AGE-BSA-stimulated osteoarthritis chondrocytes

**Figure 6:**
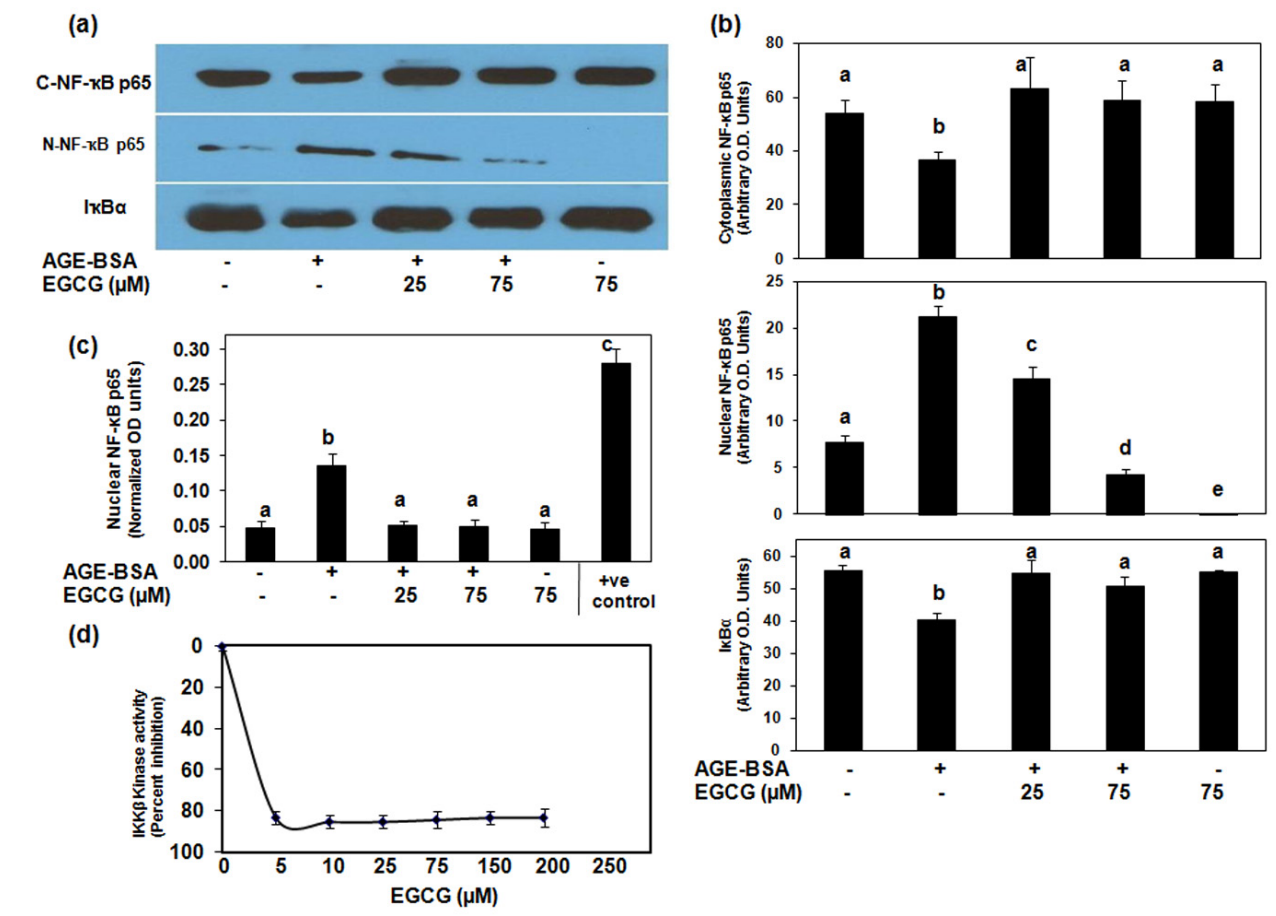
Epigallocatechin-3-gallate inhibits activation and DNA binding of NF-κB in advanced glycation end product-BSA-stimulated osteoarthritis chondrocytes. Primary chondrocytes (70 to 80% confluent) were pretreated with epigallocatechin-3-gallate (EGCG) (25 and 75 μM) for 2 hours and were stimulated by advanced glycation end product (AGE)-BSA (600 μg/ml). **(a) **IκBα degradation and NF-κB translocation were analyzed by western immunoblotting using antibodies specific for the NF-κB p65 (Santa Cruz Biotech) (C-NF-κB, cytoplasmic NF-κB; N-NF-κB, nuclear NF-κB) and for IκBα (Santa Cruz Biotech). **(b) **Band intensities were obtained as described above. Data shown are cumulative of three experiments, and the optical density values (pixels/band) presented as mean ± standard deviation. **(c) **Activated NF-κB p65 in the nucleus was determined by the highly specific Transcription Factor ELISA kit (Panomics). The positive control nuclear extract supplied with the kit was used. Data are representative of two experiments and presented as mean ± standard deviation; data without a common letter differ, *P *< 0.05. **(d) **EGCG inhibited the IKKβ kinase activity *in vitro*. IKKβ kinase activity was determined in the absence or presence of EGCG (5 to 200 μM) using the HTScan^® ^IKKβ Kinase Assay Kit (Cell Signaling Technology). Each point is representative of three individual kinase assays and presented as mean ± standard deviation.

NF-κB is an important transcriptional regulator of inflammatory cytokine gene expression and plays a crucial role in immune and inflammatory response. After the ubiquitination and phosphorylation of IκBα, the inhibitor is degraded and the NF-κB is translocated to the nucleus, where it binds and activates the promoter of target genes. To investigate the mechanism responsible for the inhibitory effect of EGCG on proinflammatory cytokine gene expression, we examined the effect of EGCG on NF-κB activation and translocation to the nucleus using western blotting. Stimulation of OA chondrocytes with AGE-BSA induced the degradation of IκBα and nuclear translocation of NF-κB p65 (Figure 6a,b). Pretreatment with EGCG (25 and 75 μM) inhibited the AGE-BSA-induced degradation of IκBα and nuclear translocation of NF-κB p65 (Figure 6a,b). To determine whether EGCG also inhibits AGE-BSA-induced DNA binding activity of NF-κB, we used the Transcription Factor ELISA kit. Exposure of OA chondrocytes to AGE-BSA significantly enhanced the DNA binding activity of NF-κB p65 compared with controls (*P *< 0.0001), and increasing doses of EGCG (25 to 75 μM) significantly reduced the AGE-BSA-induced DNA binding activity of NF-κB p65 (*P *< 0.05) (Figure 6c).

To further strengthen the relation of inhibition of the NF-κB pathway and the expression of TNFα in our studies, we next investigated the effect of a pharmacological agent (MG-132, a known inhibitor of NF-κB) on the expression of TNFα and MMP-13. Treatment of chondrocytes with the proteasome inhibitor MG-132 (100 μM) significantly blocked the AGE-BSA-induced TNFα expression (*P *< 0.05) (Figure 3a), whereas MMP-13 mRNA expression was also inhibited by MG-132 but the inhibition was not statistically significant (*P *> 0.05) (Figure 4a). Together these results suggest that EGCG exerts its inhibitory effect on TNFα expression in AGE-BSA-stimulated OA chondrocytes via modulation of the activation and DNA binding activity of NF-κB.

### Inhibition of IKKβ kinase activity by EGCG

The effect of EGCG on the phosphorylating activity of IKKβ kinase was determined using an HTScan^® ^IKKβ Kinase Assay Kit (Cell Signaling Technology). Purified IKKβ kinase was pretreated with different doses of EGCG (5 to 200 μM) 5 minutes prior to incubation with the substrate peptide. Figure 6d shows that IKKβ kinase activity was significantly inhibited by EGCG treatment (*P *< 0.001). A maximum of 85% inhibition of enzymatic activity was observed with 5 μM EGCG, after which no significant inhibition (*P *> 0.05) of enzyme activity was observed (Figure 6d). These data suggest that EGCG suppresses the activation of NF-κB by inhibiting the enzyme activity of IKK complex.

## Discussion

Chondrocytes are the only cellular components of cartilage. Under normal physiologic conditions, chondrocytes maintain an equilibrium between anabolic and catabolic activities that is necessary for preservation of the structural and functional integrity of the tissue. Chondrocytes express inflammatory mediators such as TNFα and proteolytic enzymes – aggrecanases and MMPs – which under normal conditions, mediate a very low matrix turnover required for cartilage remodeling [45]. However, in pathologic conditions such as OA, however, chondrocyte production of these inflammatory mediators and enzymes increases considerably, resulting in cartilage destruction [46].

Age is by far the most important risk factor for the development of OA [45]. By which mechanism aging is involved in the development of this debilitating disease remains largely unknown. Fatigue failure of the cartilage collagen network due to repetitive loading has long been recognized as one of the mechanisms involved in the development of OA [46]. With increasing age, the strength of the collagen matrix to withstand loading diminishes. Age-related changes in articular cartilage that influence the composition and strength of the cartilage matrix are therefore very probably involved in the development of OA [47,48]. One such change, the age-related accumulation of AGEs, has previously been shown to increase tissue stiffness, to decrease extracellular matrix turnover (synthesis and degradation), and to affect many cellular processes [17]. It is well documented that human chondrocytes are highly responsive to AGEs [18-20], and the most striking effect of AGEs or AGE-BSA on chondrocytes is to induce the production of TNFα [18] and MMP-13 [18,19], which are important sources of inflammation and cartilage degradation within the arthritic joints.

Although arthritis is present in every population and OA is the most common joint disorder, treatment is still limited to a few classes of drugs, primarily nonsteroidal anti-inflammatory drugs and corticosteroids [49,50]. While providing relief from pain, however, none of these agents has been shown to inhibit cartilage breakdown or to inhibit disease progress; they also have varying degrees of gastrointestinal toxicity [51]. Previous studies from our laboratory have shown that green tea inhibited the development of arthritis in a mouse model and also inhibited the production of various inflammatory mediators by human chondrocytes stimulated with IL-1β [30-34]. Studies from other investigators have shown that EGCG inhibits the degradation of human cartilage proteoglycan and type-2 collagen [52] and selectively inhibits the ADAMTS-1, ADAMTS-4, and ADAMTS-5 [53]. In the present study, we determined the effect of EGCG on the induction of the major proinflammatory mediators TNFα and MMP-13 in AGE-BSA-stimulated human OA chondrocytes. Almost complete inhibition of both TNFα and MMP-13 expression and production was observed at a concentration of 75 μM EGCG (*P *< 0.01) – although these concentrations of EGCG may not be achieved physiologically through oral consumption but may readily be achieved through local administration. Our results presented here demonstrate that EGCG is a potent inhibitor of AGE-BSA-induced expression and production of these inflammatory mediators in human chondrocytes.

The signaling pathways characterized by the MAPKs p38, ERK, and JNK are known to play a potential role in the regulation of inflammatory response [26,54]. These are the key players in the molecular and cellular events associated with the pathogenesis of inflammatory arthritis and are being studied as a rational target for arthritis therapy [54]. The activation of RAGE stimulates critical signaling pathways linked to inflammation, resulting in the activation of various inflammatory genes [55]. The interaction of MAPKs and RAGE has been well reported [17]. In the present study we found that EGCG specifically inhibited the AGE-BSA-induced activation of MAPKs and inhibited the expression of TNFα and MMP-13 by human OA chondrocytes. In addition, the p38-specific, JNK-specific and ERK-specific inhibitors SB202190, SP600125 and PD98059 also reduced TNFα gene and MMP-13 expression in human chondrocytes. These data suggest that EGCG has the potential to inhibit the inflammatory stimuli-induced MAPK activation and the downstream TNFα and MMP-13 gene and protein expression.

Activation of the master transcription factor NF-κB leads to the coordinated expression of many genes that encode cytokines, chemokines, enzymes, and adhesion molecules involved in mediator synthesis, and the further amplification and perpetuation of the inflammatory reaction [42,43]. The NF-κB transcription factors are present in the cytosol in an inactive state, complexed with the inhibitory IκB proteins [56]. Activation of NF-κB is a common pathway based on the induction of phosphorylation, which mediates proteasomal degradation of IκB [56]. The key regulatory step in this pathway involves activation of a high-molecular-weight IKK complex, whose catalysis is generally carried out by three tightly associated IKK subunits. IKKα and IKKβ serve as the catalytic subunits of the kinase. IKKγ serves as the regulatory subunit [57]. Activation of IKK depends on phosphorylation; serines 177 and 181 in the activation loop of IKKβ (176 and 180 in IKKα) are the specific sites whose phosphorylation causes conformational changes resulting in kinase activation [58]. It is also well documented that NF-κB is known to be involved in AGE-mediated effects of RAGE signaling [17], and that expression of TNFα and MMP-13 gene is dependent on the activation of transcription factor NF-κB [17,59].

Suppression of NF-κB activation has been linked with anti-inflammatory activity; we therefore postulated that EGCG mediates its inhibitory effects on TNFα and MMP-13 expressions, at least in part, through the suppression of NF-κB activity. In AGE-BSA-stimulated human OA chondrocytes, EGCG inhibited the degradation of IκBα and nuclear translocation of the NF-κB p65 (Figure 6a,b). In addition, DNA binding activity of nuclear NF-κB p65, as demonstrated by highly sensitive and specific ELISA, was also inhibited in OA chondrocytes. These data further confirm that EGCG attenuates the inflammatory stimuli-induced activation and DNA binding activity of NF-κB in human chondrocytes. In order to gain further insight into the mechanism, we used an *in vitro *kinase activity assay. Our results showed that EGCG inhibited the phosphorylating activity of IKKβ kinase, indicating that the observed inhibition of NF-κB in the above studies may have been achieved by inhibition of the IKK activity in OA chondrocytes, causing IκBα to accumulate in the nucleus.

In the present article we report for the first time that EGCG, the most abundant and biologically active catechin of green tea, inhibits the inflammatory activity of AGE-BSA-stimulated human OA chondrocytes. This is achieved by blocking MAPKs and NF-κB activation in human chondrocytes. Our results also point out that inhibition of NF-κB was achieved by inhibiting the degradation of the inhibitor IκBα in the cytoplasm of human OA chondrocytes as previously reported [31]. As TNFα and MMP-13 genes are NF-κB dependent, inhibition of NF-κB also inhibits their expression and production in AGE-BSA-stimulated chondrocytes.

There are a number of studies documenting the beneficial health effects of green tea consumption. Most of these studies place emphasis on the anticancer properties of green tea [27-29], which have now been attributed, at least in part, to the ability of green tea polyphenols to inhibit the inflammatory processes [30]. To this, based on our results, we can add that EGCG is a potent inhibitor of AGE-BSA-induced induction of TNFα and MMP-13 at a physiologically achievable concentration (25 μM; see Figures 3a and 4a) – but for more complete inhibition higher dose is needed. We therefore conclude that inhibition of arthritis following green tea consumption in an animal model [30] and inhibition of cartilage degradation and production of inflammatory mediators by EGCG may be the result of inhibition of some of the matrix-degrading enzymes/factors at the mRNA level through inhibition of NF-κB. It is therefore tempting to suggest that green tea polyphenol EGCG or compounds derived from it may serve as lead agents in the design of more potent and effective inhibitors of proinflammatory cytokines and collagenases for use therapeutically to block cartilage degradation in OA.

## Conclusions

The present article is the first report that shows green tea catechin EGCG inhibits the inflammatory activity against AGE-induced activation of human OA chondrocytes. The results of the present study indicate that EGCG inhibits AGE-BSA-induced upregulation of TNFα and MMP-13 viainhibiting the MAPK and NF-κB activation in human OA chondrocytes. EGCG or EGCG-derived compounds may be of value for the treatment of inflammatory arthritis in which AGEs play an active role.

## Abbreviations

AGE: advanced glycation end product; bp: base pairs; BSA: bovine serum albumin; EGCG: epigallocatechin-3-gallate; ELISA: enzyme-linked immunosorbent assay; FCS: fetal calf serum; H & E: hematoxylin and eosin; IKK: IκB kinase; IL: interleukin; MAPK: mitogen-activated protein kinase; MMP: matrix metalloproteinase; NF: nuclear factor; OA: osteoarthritis; PBS: phosphate-buffered saline; PCR: polymerase chain reaction; RAGE: receptor for advanced glycation end products; RT: reverse transcriptase; TNF: tumor necrosis factor.

## Competing interests

The authors declare that they have no competing interests.

## Authors' contributions

ZR carried out the experimental work, data collection and interpretation, and manuscript preparation. ANA, NA, SR, and FRV carried out the experimental work and collection of data. TMH conceived of the study design, coordinated the studies, data interpretation and manuscript preparation. All authors have read and approved the final manuscript.
